# Comparative Binding Study of Gliptins to Bacterial DPP4-like Enzymes for the Treatment of Type 2 Diabetes Mellitus (T2DM)

**DOI:** 10.3390/ijms25115744

**Published:** 2024-05-25

**Authors:** Laureano E. Carpio, Marta Olivares, Alfonso Benítez-Paez, Eva Serrano-Candelas, Stephen J. Barigye, Yolanda Sanz, Rafael Gozalbes

**Affiliations:** 1 ProtoQSAR SL, CEEI (Centro Europeo de Empresas Innovadoras), Parque Tecnológico de Valencia, 46980 Valencia, Spain; lcarpio@protoqsar.com (L.E.C.); eserrano@protoqsar.com (E.S.-C.); 2 MolDrug AI Systems SL, 46018 Valencia, Spain; 3 Microbial Ecology, Nutrition and Health Research Unit, Institute of Agrochemistry and Food Technology, Spanish National Research Council (IATA-CSIC), 46980 Valencia, Spain; m.olivares@iata.csic.es (M.O.); abenitez@iata.csic.es (A.B.-P.); yolsanz@iata.csic.es (Y.S.)

**Keywords:** DPP4, microbiome, molecular modeling, type 2 diabetes, gliptins

## Abstract

The role of the gut microbiota and its interplay with host metabolic health, particularly in the context of type 2 diabetes mellitus (T2DM) management, is garnering increasing attention. Dipeptidyl peptidase 4 (DPP4) inhibitors, commonly known as gliptins, constitute a class of drugs extensively used in T2DM treatment. However, their potential interactions with gut microbiota remain poorly understood. In this study, we employed computational methodologies to investigate the binding affinities of various gliptins to DPP4-like homologs produced by intestinal bacteria. The 3D structures of DPP4 homologs from gut microbiota species, including *Segatella copri*, *Phocaeicola vulgatus*, *Bacteroides uniformis*, *Parabacteroides merdae*, and *Alistipes* sp., were predicted using computational modeling techniques. Subsequently, molecular dynamics simulations were conducted for 200 ns to ensure the stability of the predicted structures. Stable structures were then utilized to predict the binding interactions with known gliptins through molecular docking algorithms. Our results revealed binding similarities of gliptins toward bacterial DPP4 homologs compared to human DPP4. Specifically, certain gliptins exhibited similar binding scores to bacterial DPP4 homologs as they did with human DPP4, suggesting a potential interaction of these drugs with gut microbiota. These findings could help in understanding the interplay between gliptins and gut microbiota DPP4 homologs, considering the intricate relationship between the host metabolism and microbial communities in the gut.

## 1. Introduction

The causes of the diabetes epidemic are multifaceted and include population aging, economic development, urbanization, Westernized dietary habits (enriched in saturated fats and refined sugars), genetic predisposition, and sedentary lifestyles [1]. Diabetes is a complex metabolic disorder characterized by high blood glucose levels due to insulin resistance, insufficient insulin secretion, or both. Hyperglycemia is the primary clinical manifestation of diabetes. However, insulin deficiency and/or resistance also lead to disruptions in lipid and protein metabolism, as well as mineral and electrolyte imbalances [2]. Most diabetic patients fall into two main categories: type 1 diabetes mellitus (T1DM), caused by a severe or near-complete lack of insulin due to genetic causes, and type 2 diabetes mellitus (T2DM), characterized by insulin resistance and inadequate compensatory insulin secretion. Additionally, there are various uncommon types of diabetes resulting from infections, drug use, hormonal disorders, pancreatic damage, or genetic defects. These distinct forms are classified separately as “Other Specific Types” [3].

Among the different diabetes classifications, T2DM is significantly more prevalent, constituting over 90% of all cases and being strongly associated with the worldwide increase in obesity [4]. In T2DM, insulin resistance is responsible for increased glucose production in the liver and decreased glucose uptake in muscle and adipose tissue at a set insulin level. Additionally, β-cell dysfunction results in reduced insulin release, insufficient to maintain normal glucose levels. Both insulin resistance and β-cell dysfunction are early pathogenic events in the development of T2DM [5].

Epidemiological and clinical studies, together with omics-based research and preclinical experiments, demonstrate the significant impact of the gut microbiota on human health and disease susceptibility [6]. This microbial ecosystem includes a wide array of interrelated bacteria, archaea, bacteriophages, eukaryotic viruses, and fungi that coexist on human surfaces and in all bodily cavities [7]. Despite significant variations in the pathophysiology of prevalent chronic metabolic disorders, there are commonalities and distinctiveness in the composition and function of the intestinal microbiota [8,9,10,11]. Regarding T2DM patients, it has been described that their gut microbiome is enriched in certain functional genes and pathways, such as sugar-related membrane transport, branched-chain amino acid outward transport, methane metabolism, xenobiotic degradation, and sulfate reduction [11]. The microbiome of individuals with overt T2DM is deficient in bacterial butyrate producers and displays an increase in species with a pro-inflammatory profile [12]. As proof of the importance of the gut microbiome in T2DM development, epidemiological studies have demonstrated that patients with total colectomy have a higher risk of T2DM than individuals without colectomy [13]. Furthermore, mechanistic studies conducted on rodents have shown that hyperglycemia can increase intestinal barrier permeability through GLUT2-dependent transcriptional reprogramming of intestinal epithelial cells, which alters tight junction integrity, ultimately resulting in leaky mucosa [14]. More recent evidence in obese mice shows the potential of the intestinal microbiota to modulate distal gut hormonal secretion with consequences in glucose regulation [15].

Drugs impact the gut microbiome, and for this reason, the associations between the gut microbiome features and T2DM in patients undergoing multiple drug treatments are challenging due to drug confounders. Among the drugs prescribed for T2DM, metformin appears to have the most significant effect on the gut microbiota, with alterations in the relative abundance of multiple genera and species and enhancement in several microbiome functional capabilities, such as propionate and butyrate production, which seems to promote intestinal gluconeogenesis [16,17,18].

**Figure 1:**
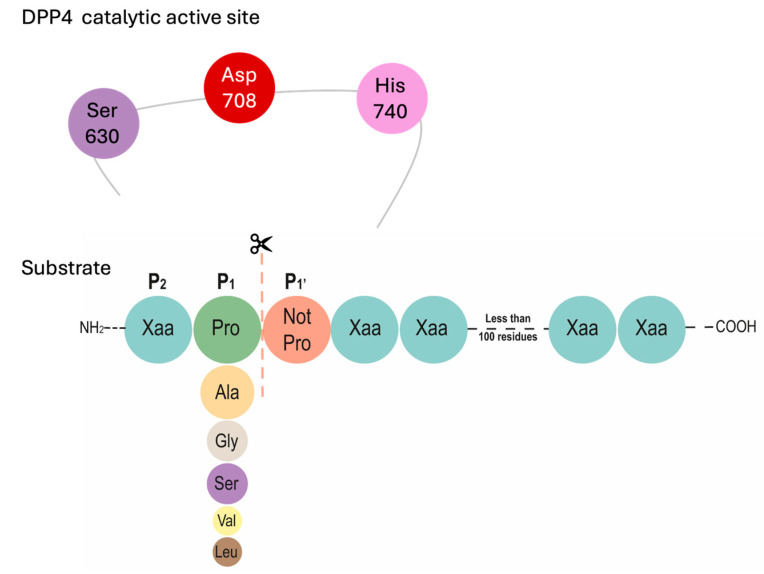
Catalytic active site residues and substrate specificity of amino peptidase DPP4. DPP4 liberates a di-peptide from the substrates. It prefers small proteins of less than 100 amino acids with a preference for proline at the penultimate N-terminal position, even though some residues such as alanine, glycine, serine, valine, or leucine can be hydrolyzed at a slower rate. This enzyme is unable to cleave substrates that present proline at N-terminal position three.

After metformin, the main drug treatments for T2DM patients are dipeptidyl peptidase 4 (DPP4) inhibitors. DPP4 is an aminopeptidase that preferentially cleaves proline or alanine residues in the penultimate position of its substrates, although it can also cleave other residues (Figure 1). It is an integral membrane protein expressed in various tissues, including intestinal and renal brush border membranes, the vascular endothelium, the liver and pancreas, glandular epithelial cells, and immune cells, and can be detected in soluble form (amino acids 49–766) in the bloodstream and other fluids (i.e., seminal fluid and cerebrospinal fluid). The protein has a large extracellular domain that is anchored to the cell membrane and contains a cysteine-rich region and one rich in glycosylation sites. The catalytic site resides in the C-terminal region, while the extracellular domain is involved in non-enzymatic functions, such as interacting with other proteins and functioning as binding sites for receptors and transporters [19,20,21,22,23].

The relationship between DPP4 and glucose homeostasis was discovered after the identification of the intestinal hormone glucagon-like peptide-1 (GLP-1) and the glucose-dependent insulinotropic polypeptide (GIP) as substrates of DPP4. The role of GLP-1 in regulating glycemia was recognized in 1986, when it was found to have significant effects on the endocrine pancreas [24,25]. In vitro kinetic studies showed that GLP-1 is a substrate for DPP4 [26]. Subsequent studies in healthy individuals and those with T2DM demonstrated that the metabolite resulting from DPP4 cleavage is the primary circulating form of GLP-1-like immunoreactivity [27] and that this metabolite is rapidly formed following exogenous administration of GLP-1 in both healthy subjects and those with T2DM [28]. Similar findings were reported in rats after exogenous GLP-1 administration [29], providing evidence that GLP-1 is a genuine physiological substrate of DPP4. These results led to the suggestion that blocking the degradation of GLP-1 by DPP4 could increase endogenous intact (active) GLP-1 concentrations and enhance its anti-hyperglycemic actions, similar to the way angiotensin-converting enzyme inhibitors are used to treat hypertension. Therefore, DPP4 inhibition was proposed as a novel therapeutic strategy for the treatment of T2DM [28,30,31].

Henceforth, the therapeutic properties of DPP4 inhibitors (DPP4is, also known as gliptins) are achieved through secondary mediation via the substrates they shield from degradation. The escalated levels of biologically active and intact GLP-1 and GIP instigate insulin and glucagon secretion in a glucose-dependent way. Moreover, DPP4is have a significant advantage over other glucose-lowering medications, as they exhibit better tolerability and safety profiles [32,33,34]. This advantage even extends to new glucose-lowering agents like sodium-glucose cotransporters II inhibitors [35,36,37].

Once DPP4 was established as a therapeutic target, comprehensive structure–activity assessments were undertaken to discover compounds that were appropriate for clinical use, leading to the development of DPP4is, such as vildagliptin [38] and saxagliptin [39]. The recognition of DPP4 as a member of an enzyme family and the elucidation of the crystal structure of DPP4 protein [40] facilitated further refinement, which culminated in the development of inhibitors such as sitagliptin [41], alogliptin [42], and linagliptin [43]. Currently, the class encompasses various DPP4is that span a spectrum of different compounds, yielding diverse chemical and pharmacokinetic profiles [44].

**Figure 2:**
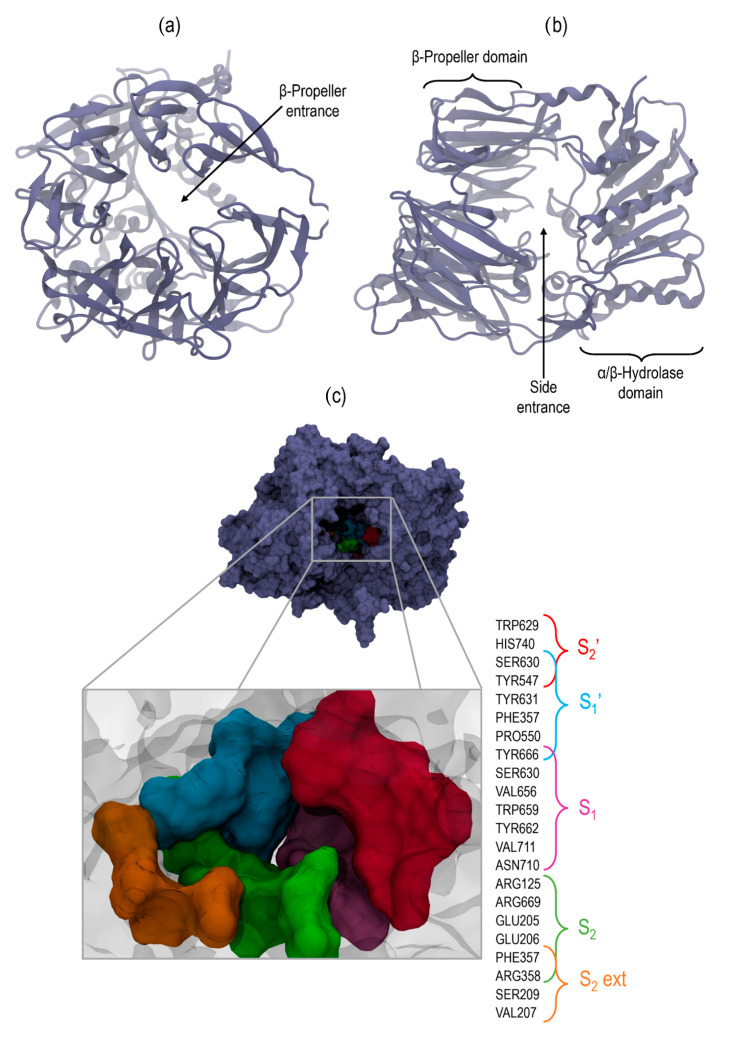
Molecular visualization of DPP4 (PDB ID: 5T4F); (**a**) β-Propeller entrance view; (**b**) Side entrance view, both in new cartoon representation and lilac color; (**c**) Binding site, with residues forming each subdomain colored in Surf representation.

The DPP4is act by targeting this enzyme binding pocket, which comprises four main regions and an additional area where known inhibitors can be attached. Therefore, DPP4is are categorized into distinct classes based on their binding locations for executing their function [45] (Figure 2). These categories are as follows:

- Class I: These attach to S_1_ and S_2_ sites; vildagliptin and saxagliptin belong to this category.
- Class II: These bind to S_1_, S_2_, S_1_′, and S_2_′ sites; alogliptin and linagliptin are part of this group.
- Class III: These interact with the S_2_ext site along with S_1_, S_2_, and S_1_′; sitagliptin is representative of this class.

While the use of gliptins has undoubtedly advanced the treatment of T2DM, further exploration in this domain remains imperative to obtain more efficient and secure gliptins. Continued research is needed to uncover innovative therapeutic options that can better address the multifaceted nature of this disease, mitigate its long-term complications, and ultimately improve the quality of life for individuals affected by this pathology. Moreover, recent studies highlighted the promise of developing therapies that target both host and gut microbial enzymes to achieve greater clinical efficacy [46] in view of the production of DPP4 activity by some gut microbiota members [23].

In this work, considering the potential role that DPP4-like isozymes produced by intestinal bacteria might play, our research endeavors to investigate the binding interactions of commercially available gliptins with various DPP4-like proteins. We employ computational methodologies to elucidate the potential interactions between these drugs and proteins produced by intestinal bacteria. Such elucidation could provide insights facilitating the development of novel and improved DPP4 inhibitors.

## 2. Results

### 2.1. DPP4 Homologs Sequence Selection and Comparison

As a first step, we worked on a selection process to identify, from human gut metagenome assemblies, different bacterial genes encoding peptides resembling human DPP4 at the amino acid sequence and protein domain organization. An array of sequences obtained from faecal samples was obtained from this step.

**Figure 3:**
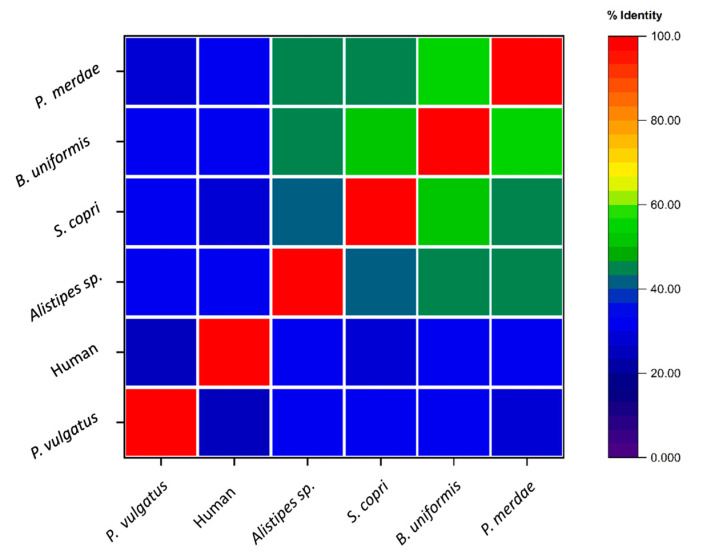
Heatmap representation of the percentage of identity between the five selected DPP4-like protein sequences and the human DPP4 sequence.

The amino acid sequence comparison allowed us to establish the degree of sequence identity among the DPP4 sequences. After analyzing this alignment data, we selected those DPP4 homologs that were relevant according to the literature but were more divergent (less than 60% sequence identity). Thus, we ultimately opted to retain the DPP4-like proteins from *Bacteroides uniformis*, *Phocaeicola vulgatus*, *Parabacteroides merdae*, *Alistipes* sp., and *Segatella copri*. Figure 3 presents the percentage identity between these homologous sequences and the human DPP4.

We also analyzed residue conservation within all the known regions of the binding pocket of human DPP4. Specifically, we scrutinized the conservation within the S_1_, S_2_, S_1_′, S_2_′, and S_2_ext zones of the binding pocket, which are considered essential for substrate interaction and enzymatic activity. Table S1 provides a representation showcasing the sequence alignment comparison of the DPP4 homologs in relation to the human reference sequence. To better understand the conservation within the binding pocket zones, each residue in these zones was color-coded based on their physicochemical properties. This approach takes into consideration not only the conservation of the specific amino acids but also their properties. Applying this color-coding approach makes it easier to identify residues that exhibit broader conservation across the DPP4 sequences that were studied.

Table S1 also shows trends regarding residue conservation across different regions within the enzyme binding pocket. Specifically, the residues found in the S_1_ and S_1_′ regions showed a higher degree of conservation than those in the S_2_, S_2_′, and S_2_ext regions. The S_2_ext region stands out due to its pronounced variability and alterations among the different DPP4-like proteins studied.

### 2.2. DPP4 Homologs Structure Prediction and Evaluation

Once we selected the sequence of bacterial DPP4 functional homologs, we proceeded with the prediction of 3D protein structures utilizing two different approaches, RosettaFold (https://robetta.bakerlab.org/, accessed on 29 April 2022) and YASARA (Version 21.6.17) homology modeling functionality. For each DPP4-like protein, the predicted structures from each approach underwent optimization, focusing on identifying the best-optimized model.

Finally, we selected the best models for a complete analysis using the Ramachandran Plot calculation within the SAVES Server. Table 1 shows the different metrics for the best structure obtained for each DPP4-like protein.

This step yielded optimal 3D structures of the DPP4-like protein of five different intestinal bacteria, showing, for each case, more than 95% of the residues falling in the most favored and allowed regions, with less than 1% falling into disallowed regions.

**Figure 4:**
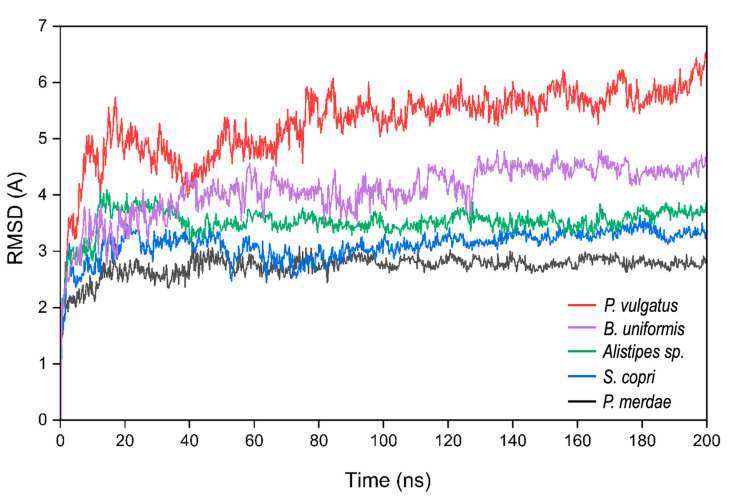
RMSD calculations for the 200 ns of simulations for all the predicted 3D structures.

In preparation for our computational work involving the predicted structures, we subjected the DPP4-like proteins to molecular dynamics (MD) simulations spanning a duration of 200 nanoseconds (ns) to ascertain the stability of the predicted structures over time, ensuring that the structural integrity remains intact. Afterward, we evaluated the root mean squared deviation (RMSD) values of the simulation (Figure 4). RMSD values offer a depiction of how each DPP4 homolog fluctuates in structure throughout the simulation trajectory, underscoring their capability to retain their conformational arrangement with time.

The RMSD plot (Figure 4) illustrates the convergence of these structures to a consistent state with time, which substantiates the stability attained by each DPP4 homolog’s structure during the 200 ns MD simulations.

In some cases, such as for *P. merdae*, *Alistipes* sp., and *S. copri*, the RMSD values exhibit minimal fluctuations, remaining within a range of less than 0.5 Å during the final 40 ns of the simulation. The case of *B. uniformis*, while presenting slightly higher RMSD values than the homologs mentioned above, follows a similar stabilization pattern. Its RMSD values exhibit fluctuations of less than 0.5 Å in the last 40 ns of the simulation, indicating a reassuring degree of structural steadiness.

Although *P. vulgatus* registers a relatively higher RMSD score, its trajectory during the last 40 ns demonstrates fluctuations within approximately 1 Å. This suggests a reasonably acceptable stabilization, even though the RMSD values are comparatively elevated.

Collectively, these observations suggest the stability of the DPP4 homolog structures after the 200 ns MD simulations. These simulations provide confidence that the predicted structures align well with the dynamic behavior of the proteins, thus making sense for subsequent computational investigations.

### 2.3. Computational Prediction of the Binding of Known Gliptins with Different DPP4s

After identifying noteworthy similarities between human DPP4 and bacterial DPP4-like proteins, a computational investigation employing a molecular docking approach was conducted. This step aimed to predict the binding of well-known commercially available gliptins—namely, sitagliptin, saxagliptin, vildagliptin, linagliptin, alogliptin, and teneligliptin—within the binding sites of all the studied DPP4-like structures. The resulting docking scores (kcal/mol) are presented in Table 2. These scores were generated by docking the gliptins into the binding pocket of human DPP4 and the suggested binding pocket indicated by the sequence alignment study for the bacterial DPP4-like proteins.

Our results showed that mean docking scores and standard deviations were quite similar between the different variants and the human DPP4. For the human variant, the mean docking score was 8.20 kcal/mol, with a standard deviation of 1.2 kcal/mol, while for the bacterial variants, we observed a mean value ranging from 7.2 to 9.1 kcal/mol. Moreover, these outcomes indicate that, among the studied bacterial DPP4-like proteins, the *P. merdae* variant tends to exhibit the highest mean docking scores for the tested gliptins. Despite slight variations in docking scores across the DPP4-like proteins, their scores are similar to the scores of the human variant. This resemblance in binding affinities suggests the potential for these gliptins to interact effectively with DPP4-like bacterial enzymes, akin to their interactions with the human DPP4.

**Figure 5:**
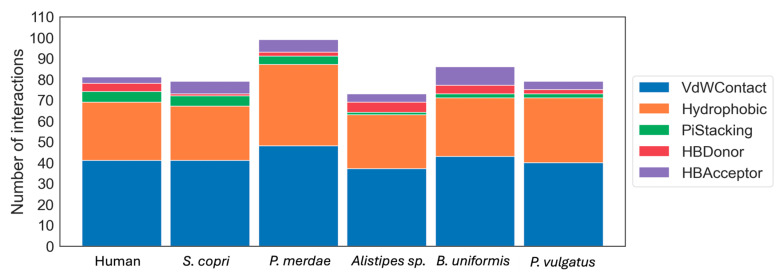
Stacked bar plot of the interaction count of the different gliptins with DPP4 homologs colored by the type of interaction.

Moreover, these best poses obtained from the docking experiments were subjected to more calculations to obtain the interactions that each drug presented with the DPP4-like proteins. The type of each interaction was quantified for all drugs in each DPP4-like enzyme binding, showing similar values not only in the number but also in the type of these interactions (Figure 5). The binding of gliptins with *P. merdae* showed a higher number of interactions, in concordance with the docking scores presented before, while *Alistipes* sp. presented the lowest number of interactions with the different gliptins. Moreover, it can be observed how the distribution of the type of interactions is maintained in the different homologs, with Van Der Waal contacts being the most common interaction, followed by hydrophobic, hydrogen bonds, and pi-stacking.

**Figure 6:**
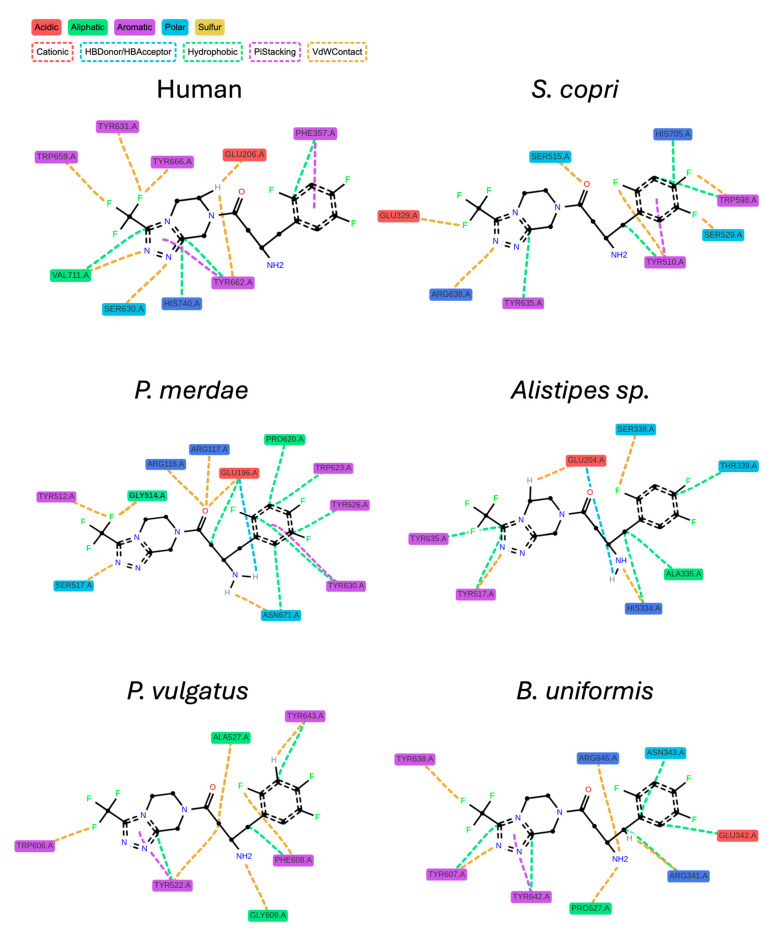
The 2D interaction maps of sitagliptin predicted best pose with the six different DPP4-like proteins studied obtained with ProLiF package. Discontinued double line in the molecule rings represents aromaticity.

Finally, 2D interaction maps of each pose were obtained, resulting in a visualization of the different interactions that occur in the predicted binding of each drug. Figure 6 shows these maps for the case of sitagliptin, as it is one of the more often administered drugs for T2DM treatment worldwide. These 2D maps allowed us to study the predicted mechanism of action of sitagliptin among the different DPP4-like proteins. All poses maintained hydrophobic interactions between the molecule and the different residues of the binding pocket; however, only the binding with *S. copri*, *P. merdae*, *P. vulgatus,* and *B. uniformis* maintained the pi-stacking interactions with one of the aromatic rings of sitagliptin, while the binding with the human DPP4 showed this type of interactions with both. The 2D interaction maps for all the gliptins studied are presented in the Supplementary Material (Figures S1–S5). Focusing on the other gliptins, it can be observed how in the case of alogliptin, all DPP4-like proteins bindings, except *B. uniformis*, maintain a pi-stacking interaction with one of the aromatic rings, as occurs with the binding with human DPP4. Linagliptin binding with all the DPP4s shows a majority of hydrophobic interactions, while in the binding of saxagliptin, only *P. vulgatus* and *B. uniformis* presented hydrogen bonds as the human DPP4 binding. For teneligliptin, only the bindings with *B. uniformis* and *P. merdae* presented a pi-stacking interaction with one of the aromatic rings, and lastly, in the case of vildagliptin, only *S. copri* and *P. vulgatus* presented hydrogen bond interactions in a similar way as the binding with the human DPP4.

## 3. Discussion

The gut microbiota residing in the gastrointestinal tract plays a crucial role in host metabolism and immune function. While gliptin research has mainly focused on human DPP4 activity, certain fungi and bacteria within the human-associated microbial communities also possess DPP4-like enzymatic activities. Given the significance of inhibiting DPP4 activity in managing T2DM, investigating the role of DPP4 homologs from gut microbes is of particular interest [47,48,49,50,51,52,53,54,55,56,57,58,59,60,61,62].

In this study, our focus centered on five distinct species of intestinal bacteria: *B. uniformis*, *P. vulgatus*, *P. merdae*, *Alistipes* sp., and *S. copri*. These bacteria’s DPP4-like functional homologous proteins have been demonstrated to exhibit in vitro DPP4 activity. To comprehend the mechanisms underlying this activity, we initiated our exploration with a sequence analysis to elucidate the resemblances between these homologs and the human variant.

Upon conducting sequence alignments, all five bacterial DPP4-like proteins shared a 20% to 40% similarity range with the human DPP4. Overall, our observations suggested that the binding pocket residues remain conserved in the microbiome variants, and the mutations of these residues do not compromise the aminoacids’ functional properties or disrupt the cavity’s environment. The maintenance of the conserved enzymatic cavity suggests that the enzymatic activity could also be retained between the variants, even though the mechanism of action could be different. These conserved regions could be related to the observation that the gut microbiome of T2DM patients treated with class I gliptins is altered [63]. Notably, the S_2_ext region stood out due to its marked variability and deviations. Indeed, the extended region’s significance lies more in how gliptins interact with the pocket rather than directly influencing DPP4 activity. Consequently, it is conceivable to find DPP4-like proteins exhibiting enzymatic activity even in cases where this region is not conserved.

This observation aligns with the DPP4 activity documented in experimental results and literature. For example, a significant reduction in DPP4 activity within cecal content and feces was observed with the commercially available DPP4 inhibitor vildagliptin in obese mice. Moreover, this inhibitor exerted an influence on the gut microbiota’s composition and its associated metabolic activity [62].

This outcome suggests that gliptin drugs potentially engage with microbiome-derived DPP-like enzymes, influencing their activity. Additionally, these drugs could be responsible of changes in the microbiota’s composition. However, the microbiota-shaping effects of gliptins in clinical studies and their additional hypoglycemic mechanism need further investigation [64]. Studying the interactions between these drugs and microbiota-associated DPP4-like proteins is pivotal for a deeper comprehension of the intricate connection between the gut microbiome and T2DM.

We developed and employed a computational workflow for structure prediction to study in silico the potential DPP4-like activity of microbiome homologs. This methodology enabled the computational exploration of 3D structures for DPP4-like bacterial products. Two distinct algorithms were employed, each contributing to the three-dimensional predictions: the homology modeling method embedded in YASARA [65] and the deep learning algorithm, RosettaFold [66].

Four of the five predicted variants showed the most optimal predictions through YASARA’s homology modeling, with RosettaFold delivering the optimal prediction only for one. The implementation of deep learning algorithms has triggered a revolution in protein structure prediction, but in our specific case, YASARA’s homology modeling exhibited superior performance compared to these new approaches. This could be attributed to the fact that RosettaFold was trained using a dataset of human protein structures rather than microbial counterparts [66]. This outcome may also be related to the results observed after the MD simulations of the predicted structures. The DPP4-like protein from *P. vulgatus*, predicted with RosettaFold, exhibited the highest RMSD values. This aligns with the results from the Ramachandran plot, where this protein showed the highest percentage of residues in disallowed regions, suggesting a less stable structure. Furthermore, considering that the algorithm was trained primarily on human data and *P. vulgatus* has a lower sequence identity with human DPP4 compared to the other DPP4-like proteins, it is plausible that the protein from *P. vulgatus* underwent greater conformational changes to achieve a more stable state during the MD simulations, which could explain this higher RMSD.

To our knowledge, these computations culminated in the generation of the first predicted 3D structures for these DPP4 homologs with optimal metrics. This resource equipped us to conduct molecular modeling investigations into the interactions between diverse molecules and these isozymes.

Considering the possible role of gliptins potentially interacting with bacterial DPP4-like proteins, we worked with the predicted 3D structures we obtained. We used these structures as a basis to conduct molecular docking experiments involving six distinct commercially available gliptins. The gliptins demonstrated similar binding affinities to both the microbiome variants and the human DPP4.

Furthermore, when we calculated the interactions between the best poses of each gliptin with the DPP4-like proteins studied, we observed a similar number of interactions among them. However, not all residues observed at the junctions with bacterial DPP4s coincide with those found in the sequential alignment between human DPP4 and bacterial counterparts. Although we did find the same types of residues present, they did not align with their equivalents in the sequential alignment. This discrepancy hints at a potential different binding mode in bacterial DPP4-like proteins, suggesting that the well-defined and known binding sections in the pocket of human DPP4 may be displaced in various bacterial functional homologs, as suggested in the literature [46,67]. Further, more detailed studies are imperative to ascertain this with greater certainty, as the possible binding of these gliptins to DPP4-like proteins does not need to be necessarily related to a functional inhibition of the protein activity. However, the possible binding of these drugs to a different target besides the human DPP4 is a remarkable result that could help to design better and more efficient gliptins.

The findings obtained in this work align with recent research by Wang et al. [46], where they observed the activity of DPP4-like proteins in various *Bacteroides* species members of the gut microbiota. These proteins seemed to play a role in regulating GLP-1 alongside the human variant. Indeed, the authors experimentally demonstrated that the known inhibitor sitagliptin could also inhibit *Bacteroides* DPP4 variants, although with a higher IC_50._ This finding aligns with our results, which indicated a possible binding between known inhibitors and DPP4 variants in *Bacteroides* spp. (but showing a less favorable binding score, which could be related to the higher IC_50_ that was reported) and other genera such as *Segatella*, *Alistipes*, and *Parabacteroides*.

Furthermore, the authors performed a screening and identified a compound (daurisoline-d4) capable of inhibiting *Bacteroides* DPP4-like protein *in vitro* and *in vivo* using experimental methods. This compound targets this specific *Bacteroides* DPP4 without affecting human DPP4, and thus, it is proposed as a treatment for T2DM alongside sitagliptin [46]. Following this line of study, our computational predictions could help support the design of new and more efficient gliptins that bind not only the human DPP4 but also some homologs produced by the gut microbiota, extending these insights not only to *Bacteroides* species but also to three more families of intestinal bacteria.

While the specific roles of these bacterial DPP4-like enzymes within the body need more experimental studies, including their potential connection with the GLP-1 cycle involving human DPP4, our predictions suggested a potential relationship between gliptins and these gut microbial isozymes. The predicted docking scores and calculated interactions raise questions about potential impacts on organismal health and microbiome dynamics. However, whether these interactions exert any consequential effects in the clinical setting requires a thorough investigation.

## 4. Materials and Methods

### 4.1. DPP4 Homologs Sequence Data

The sequences of the intestinal homologs of DPP4 were studied from human fecal samples collected from a previous clinical trial on overweight subjects [68]. Paired-end fastq files, depleted from human DNA reads (using conventional bowtie + samtools + bedtools algorithms and hg38 assembly), were used to assemble the fecal metagenome of each individual at week 0 and week 12, conducted on 30 overweight and obese individuals (body mass index of 25 to 40 kg/m^2^). The data were analyzed by using Velvet assembler v1.2.10 [69] with the parameters as follows: k-mer length 61, -exp_cov auto, -ins_length 250, and -ins_length_sd 60. This step was followed by an assembly refinement step using the Metavelvet extension [70] with the following parameters: -ins_length 250 -ins_length_sd 60 configuration. The assembled contigs larger than 500 nt in length were retained, and the prediction of potential ORF encoded in such fragments from respective metagenomes was assisted by FragGeneScan v1.30 [71], with the -complete = 0 and -train = complete configuration. Peptide sequences (with length ≥ 50 aa) obtained from the ORF prediction in all metagenomes were concatenated and clustered at 70% sequence identity using cdhit algorithm with -c 0.7, -G 1, -B 1, and -g 1 parameters [72]. Representative sequences from clusters were mapped against the non-redundant peptide database derived from the latest version of the assembled and annotated human genome (hg38). For such aim, we used the usearch v8.0.1623 algorithm [73] with the following parameters: -usearch_local, -id 0.7, -strand both, and -top_hit_only. Domain architecture of selected gut microbiome hits (e.g., Pfam, SMART, Interpro) as well as their molecular function (e.g., KEGG, COG, eggNOG) were evaluated to provide further evidence about their mimicked functionality. The taxonomy identification of ORFs mimicking human DPP4 function was achieved via tBlastn server (https://blast.ncbi.nlm.nih.gov/Blast.cgi, accessed on 29 April 2018) using peptide queries versus non-redundant nucleotide collection.

### 4.2. Data Retrieval

For the molecular modeling experiments conducted in this work, the three-dimensional structure of DPP4 was obtained from the Protein Data Bank (https://www.rcsb.org/, accessed on 27 February 2022) (PDB ID: 5T4F, resolution 1.9 Å). The different gliptins employed in this work, namely, sitagliptin, saxagliptin, vildagliptin, linagliptin, alogliptin, and teneligliptin, were obtained from PubChem database (https://pubchem.ncbi.nlm.nih.gov/, accessed on 29 September 2022) [74].

### 4.3. Sequence Alignment

Sequence alignments play a crucial role when working with protein sequences. By aligning sequences, similarities and differences can be identified, aiding in the prediction of protein structures, functional domains, and conserved regions. They provide insights into the structural, functional, and evolutionary relationships among proteins.

The sequence alignments for all protein sequences within this study were conducted using the Clustal Omega web server (https://www.ebi.ac.uk/jdispatcher/msa/clustalo, accessed on 29 September 2021) [75], employing its default parameters.

### 4.4. Computational Prediction of DPP4 Homologs’ 3D Structure

Computational prediction of protein structure is crucial when the 3D structure is not yet elucidated. This step allows the generation of the structure necessary for subsequent molecular modeling steps.

Initially, the sequences of different DPP4 homologs were used as input for the structure prediction methods. Two different approaches were employed for this purpose, one based on deep learning algorithms, called RosettaFold [66], and one based on YASARA homology modeling algorithm [65].

Next, the predicted structures from these different methods were assessed for quality and accuracy by the corresponding metrics. The best-predicted structure for each DPP4 homolog was further refined using GalaxyRefine via the GalaxyWeb server (https://galaxy.seoklab.org/cgi-bin/submit.cgi?type=REFINE, accessed on 29 April 2022) [76], a tool known for improving structural accuracy by performing repeated structure perturbation and subsequent overall structural relaxation by MD simulation, obtaining five refined models for each predicted structure.

**Figure 7:**
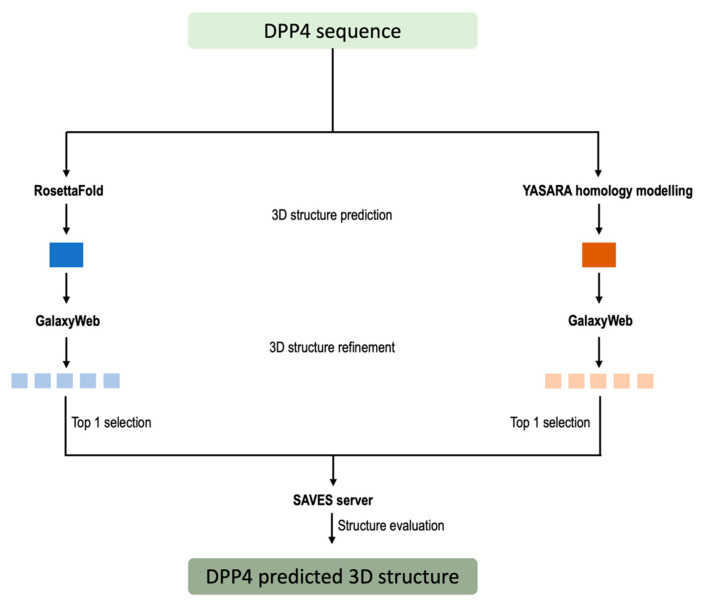
Schematic representation of the developed pipeline for the homolog 3D structure prediction.

Subsequently, the top-ranked refined structures obtained from the methods employed for each DPP4 homolog were subjected to evaluation using the SAVES Server [77] (https://saves.mbi.ucla.edu/, accessed on 29 April 2022). This evaluation involved generating Ramachandran Plots [78], which provide insights into the stereochemical quality of the protein structures. Based on the evaluation results, the best-predicted structure for each DPP4 homolog was selected as the reference structure for subsequent MM studies (Figure 7). This reference structure serves for further investigations, allowing for in-depth exploration of functional aspects and interactions of the DPP4 homologs.

### 4.5. Molecular Dynamics Simulation

The MD simulation provided information about the conformational changes, stability, and interactions of the molecules under investigation. The trajectories obtained from the simulations were analyzed and interpreted, obtaining the RMSD parameter using MAESTRO functionalities.

The MD simulations were performed with OPLS4 forcefield, establishing a timestep of 2 femtoseconds and a simulation cell of 10 Å of spacing in each coordinate axis and filling the simulation cell with TIP3P water molecules, the necessary ions to neutralize the system, and a concentration of NaCl of 0.15 M. NPT ensemble class was employed with temperature at 300 K and pressure at 1.01325 bar.

### 4.6. Molecular Docking Calculation and Analysis

In the molecular-docking process, the best binding pose of a ligand with a target protein is predicted. In this study, the YASARA [65] macro designed for this purpose was utilized with standard parameters. This macro incorporates AutoDock Vina [79] for the docking calculations. Initially, the receptor, i.e., the DPP4 protein, underwent cleaning and optimization using the YASARA software, ensuring a refined structure for docking experiments.

Subsequently, the different compounds were docked to the binding site of DPP4, employing a grid box with dimensions encompassing 5 Å around the binding pocket amino acids (selecting the residues that form the S_1_, S_1_′, S_2_, S_2_′, and S_2_ext sections) and 25 runs. Moreover, the ligand was set free while maintaining rigid the binding pocket amino acids. This focused docking approach aimed to explore potential binding interactions and orientations of the small molecules within the active site of DPP4.

After the docking simulations, the results were analyzed using YASARA software, allowing for the evaluation of binding scores and the generation of PDB files of the best pose.

Best pose of each docking score was then studied by in-house developed script that incorporated ProLiF package (version 1.0.0) [80] in Python (version 3.9.4) [81] for interaction calculation and visualization.

## 5. Conclusions

In conclusion, this study sheds light on the role of gut microbiota-derived DPP4-like functional homologs in host metabolism, particularly in the context of T2DM treatment. By focusing on five distinct species of intestinal bacteria (*Bacteroides uniformis*, *Phocaeicola vulgatus*, *Parabacteroides merdae*, *Alistipes* sp., and *Segatella copri*), this research elucidates the structural and functional similarities between bacterial DPP4-like proteins and their human counterpart. This study employed computational methods to predict 3D structures and conducted molecular docking experiments to explore the interactions between gliptin drugs and DPP4-like enzymes. The results suggest potential binding affinities between gliptins and both bacterial and human DPP4, albeit with some variations in binding modes observed in bacterial DPP4s.

These findings align with previous research indicating the inhibitory effects of gliptins on *Bacteroides* DPP4-like proteins and could help in the designing of more efficient gliptins targeting both human- and microbiota-derived DPP4 functional homologs. However, further experimental studies are warranted to elucidate the specific roles of these homologs in the GLP-1 cycle and their potential implications for organismal health and microbiome dynamics. This research contributes to exploring novel therapeutic strategies for T2DM management by considering the intricate interplay between the gut microbiota and host metabolism.

## Figures and Tables

**Table 1:** Ramachandran plot metrics for the best prediction of each DPP4 homolog alongside the algorithm employed for its generation.

DPP4 Homolog	% Most Favored Regions	% Allowed Regions	% Generally Allowed Regions	% Disallowed Regions	Algorithm
*S. copri*	92	7.4	0.3	0.3	YASARA
*Alistipes* sp.	87.5	11.7	0.5	0.3	YASARA
*B. uniformis*	90.1	9.4	0.3	0.2	YASARA
*P. merdae*	92.2	7.2	0.3	0.3	YASARA
*P. vulgatus*	91.9	6.9	0.5	0.8	RosettaFold

**Table 2:** Docking score values (kcal/mol) for the studied DPP4 variants. Higher values indicate stronger binding affinity.

Gliptin	Human	*S. copri*	*P. merdae*	*Alistipes* sp.	*B. uniformis*	*P. vulgatus*
Sitagliptin	9.23	7.92	9.01	7.45	8.23	7.87
Linalgliptin	9.91	8.92	10.80	8.69	9.64	8.62
Alogliptin	7.59	7.46	9.01	6.99	7.59	7.53
Teneligliptin	7.77	7.66	9.02	7.30	8.42	7.52
Vildagliptin	6.90	7.22	8.43	6.38	7.77	6.88
Saxagliptin	7.57	7.22	8.2	6.3	7.93	6.94
Average	8.20 ± 1.2	7.7 ± 0.6	9.1 ± 0.9	7.2 ± 0.9	8.3 ± 0.7	7.6 ± 0.6

## Data Availability

The original contributions presented in the study are included in the article/Supplementary Material, further inquiries can be directed to the corresponding author.

## Supplementary Materials

The supporting information can be downloaded at: https://www.mdpi.com/article/10.3390/ijms25115744/s1.
