# Supplementary material for: Comparative Binding Study of Gliptins to Bacterial DPP4-like Enzymes for the Treatment of Type 2 Diabetes Mellitus (T2DM)
Source: Int J Mol Sci. 2024 May 25;25(11):5744. doi: 10.3390/ijms25115744 (PMC11171585; doi:10.3390/ijms25115744)
Supplement: Supplementary file 1 [file ijms-25-05744-s001.zip › ijms-3014387-supplementary.pdf]

Supplemental information

Comparative binding study of gliptins to bacterial DPP4-like enzymes for the treatment of type 2 diabetes mellitus (T2DM).

Laureano E. Carpio [1,2], Marta Olivares[3], Alfonso Benítez-Paez[3], Eva Serrano-Candelas[1], Stephen J. Barigye[2,a], Yolanda Sanz[3], Rafael Gozalbes[1,2*]

[1] ProtoQSAR SL, CEEI (Centro Europeo de Empresas Innovadoras), Parque Tecnológico de Valencia, Spain
[2] MolDrug AI Systems SL, Valencia, Spain
[3] Microbial Ecology, Nutrition and Health Research Unit, Institute of Agrochemistry and Food Technology, Spanish National Research Council (IATA-CSIC), Valencia, Spain
[*] Correspondence: rgozalbes@protoqsar.com;
[a] Current affiliation: Xenon Pharmaceuticals, Gilmore Way, Burnaby BC, Canada.

Table S1. Comparative analysis of residues within binding pocket sections across 5 DPP4 homologs and the human variant. Colors visually represent aminoacids properties: pink indicates non-polar aliphatic residues, green represents polar uncharged residues, orange denotes aromatic R-groups, red signifies positively charged residues, and blue indicates negatively charged residues.

Human	*P. vulgatus*	*Alistipes sp.*	*S. copri*	*P. merdae*	*B. uniformis*
			**S_{2ext}**		
**VAL207**	-	-	-	-	-
**SER209**	GLY189	ALA177	ALA181	ALA188	SER195
**ARG358**	HIS323	ASP307	VAL321	TRP320	VAL335
			**S_2**		
**ARG125**	-	ARG95	ARG101	ARG105	ARG105
**PHE357**	GLN322	ALA306	GLU320	GLU319	ASN334
**GLU205**	GLN186	GLU174	GLU178	GLU186	GLU192
**GLU206**	GLU187	GLU175	GLU179	GLU187	GLU193
**ARG669**	ARG644	ILE609	ARG629	ARG622	ARG636
			**S_2'**		
**TRP629**	TRP606	TRP569	TRP589	TRP582	TRP596
**SER630**	SER607	SER570	SER590	SER583	SER597
**HIS740**	ARG727	ARG689	GLN710	ARG704	ARG718

**Human**	*P. vulgatus*	*Alistipes sp.*	*S. copri*	*P. merdae*	*B. uniformis*
			**S_1**		
**VAL656**	VAL633	VAL596	PRO616	PRO609	VAL623
**TRP659**	TRP636	TRP599	TYR619	TRP612	TRP626
**TYR662**	TYR639	TYR592	TYR622	TYR615	TYR629
**VAL711**	CYS685	VAL648	VAL667	VAL661	VAL675
**ASN710**	THR684	ASN647	ASN666	ASN660	ASN674
			**S_1'**		
**TYR547**	TYR522	TYR488	TYR501	TYR501	TYR515
**PRO550**	PRO525	PRO491	PRO504	PRO504	PRO518
**TYR631**	PHE608	TYR571	TYR591	TYR584	TYR598
**TYR666**	TYR641	TYR606	TYR626	TYR619	TYR633

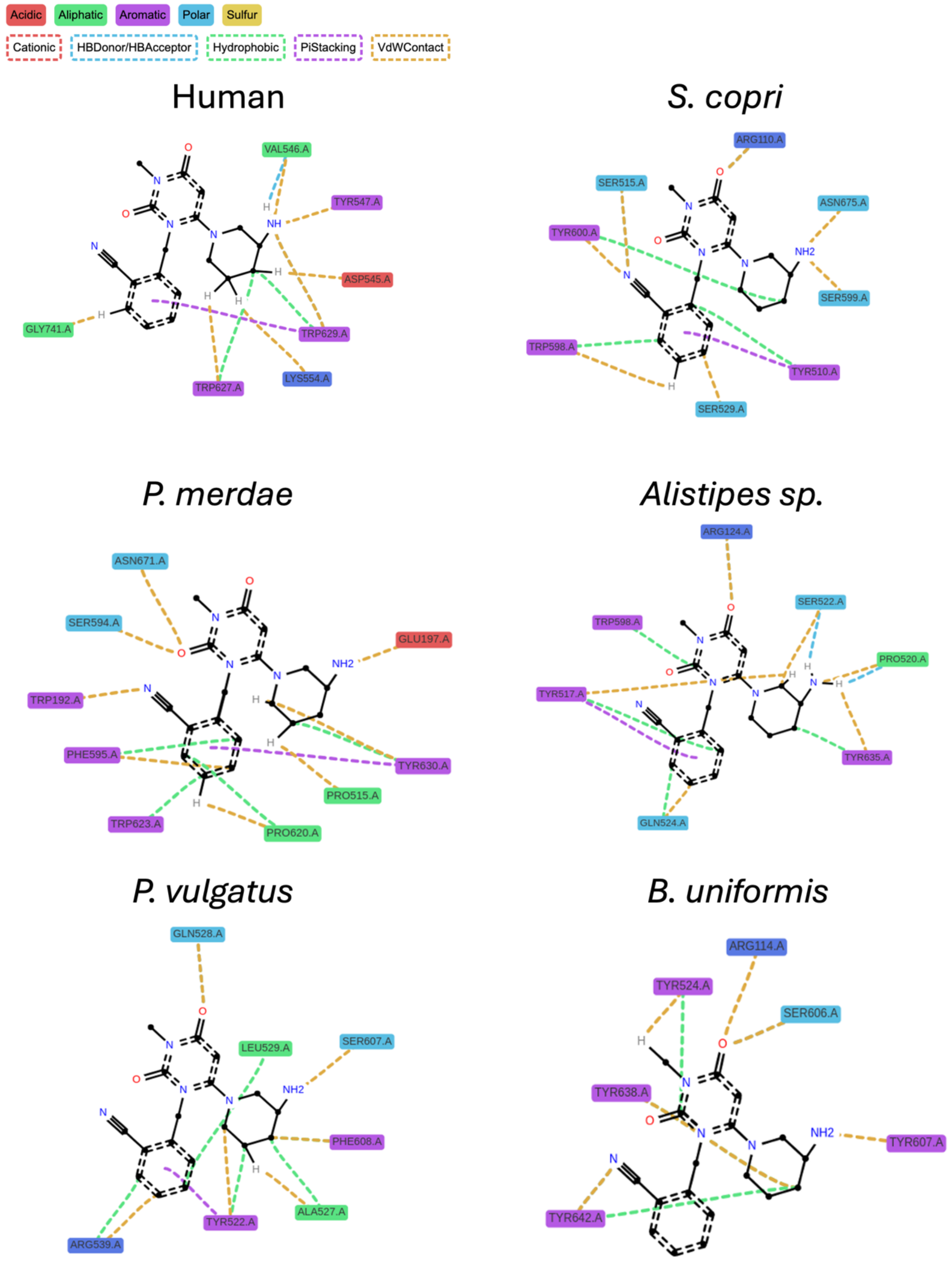

Figure S1. 2D interaction maps of alogliptin predicted best pose with the six different DPP4 homologues studied.

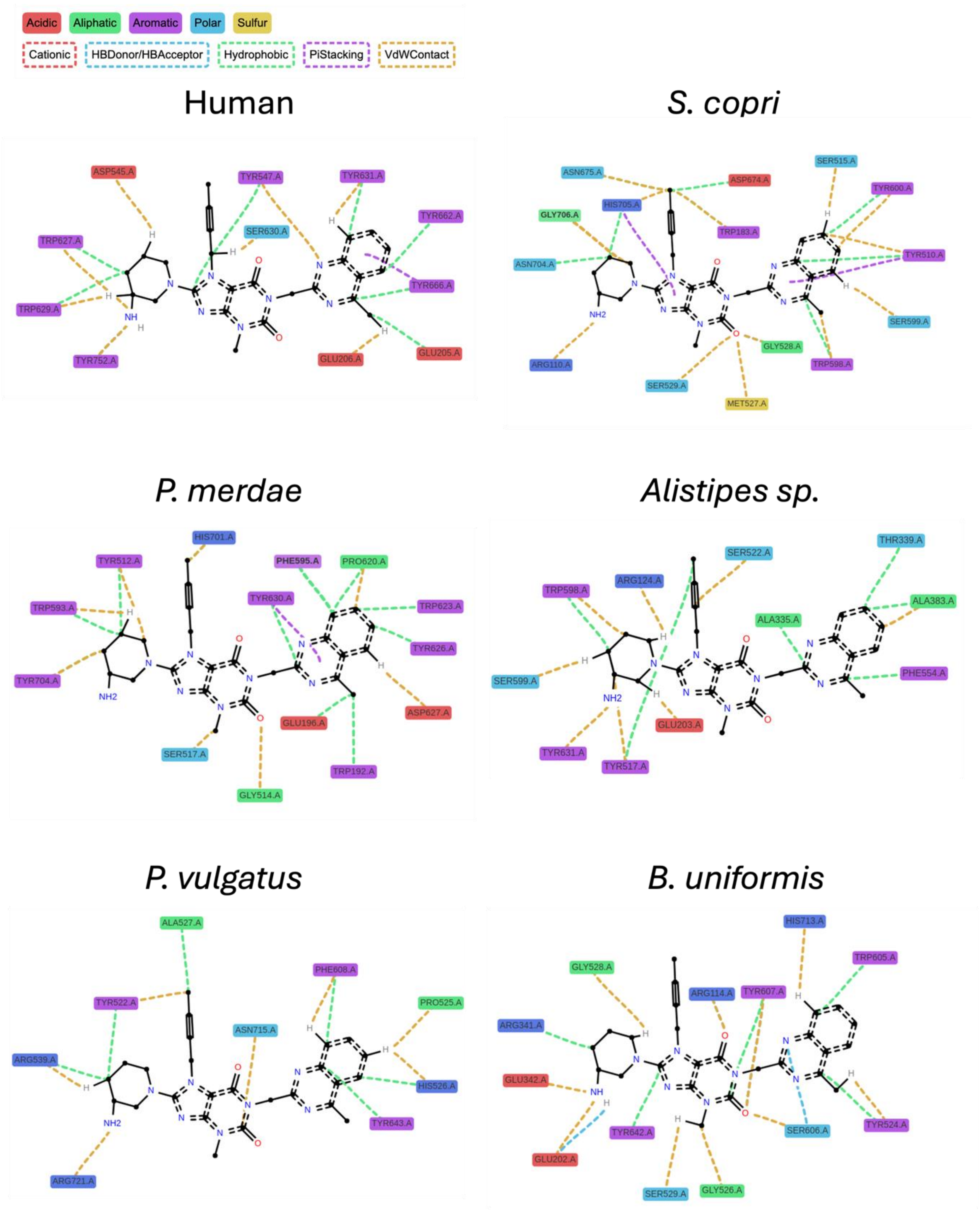

Figure S2. 2D interaction maps of linagliptin predicted best pose with the six different DPP4 homologues studied.

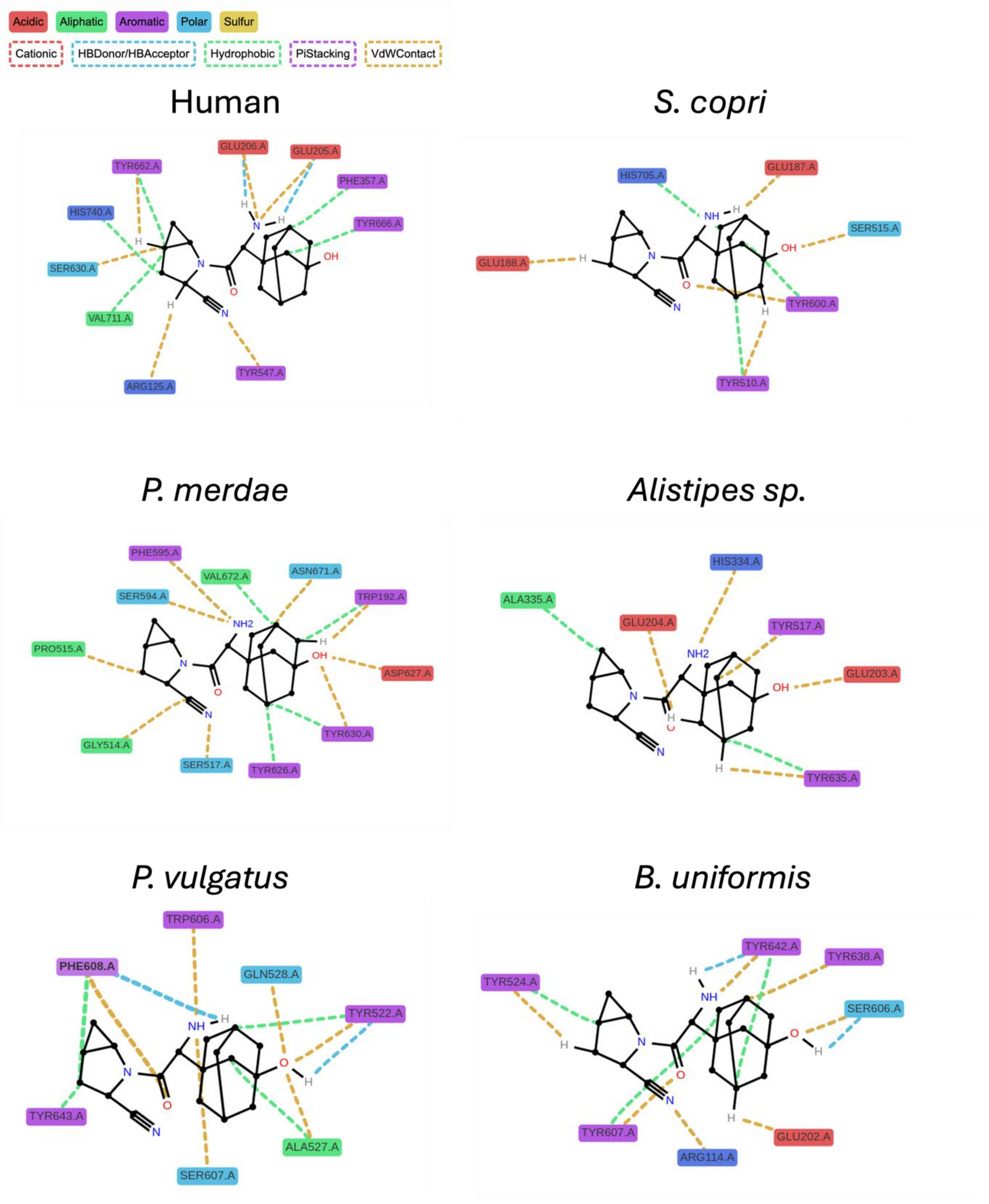

Figure S3. 2D interaction maps of saxagliptin predicted best pose with the six different DPP4 homologues studied.

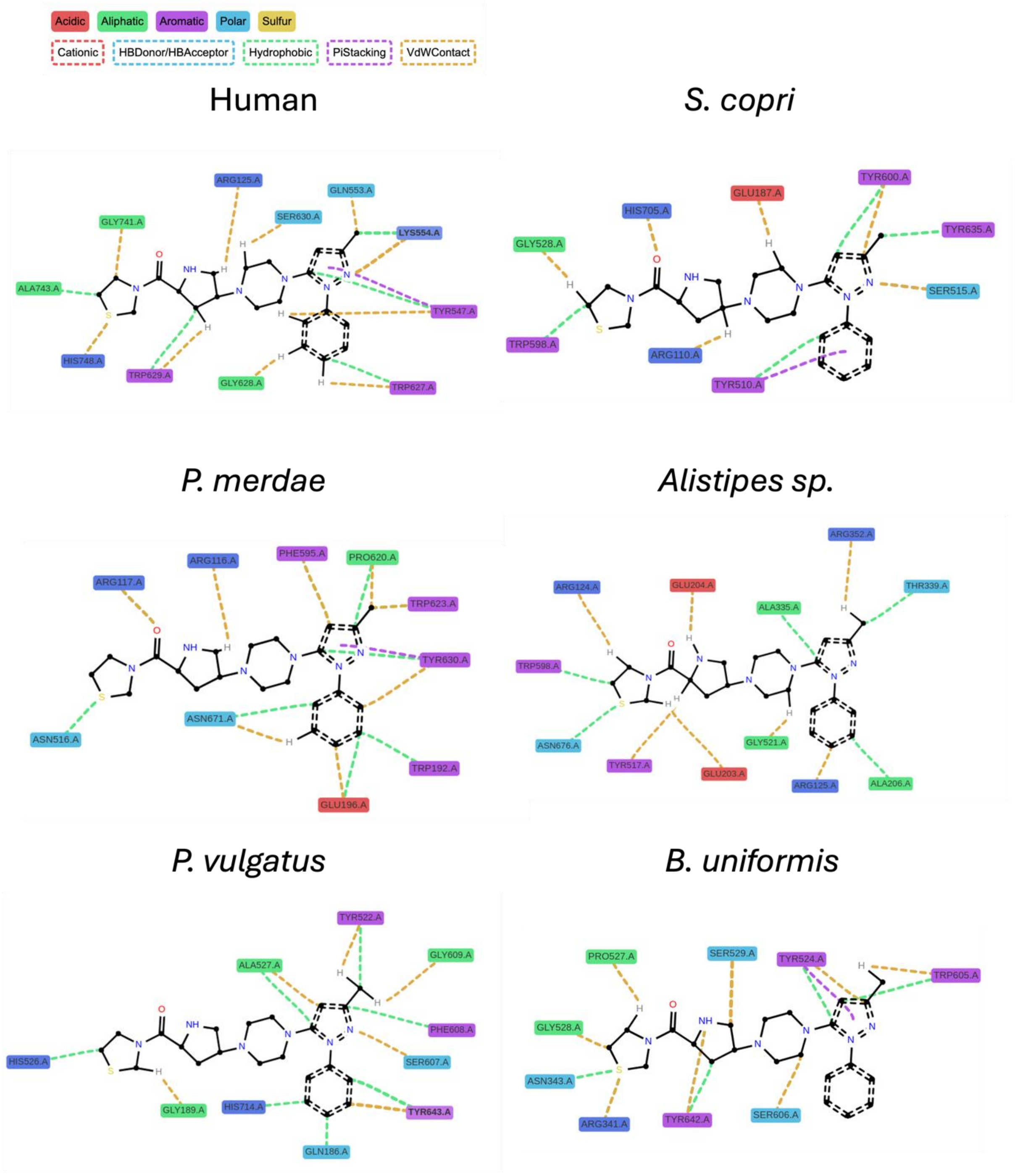

Figure S4. 2D interaction maps of teneligliptin predicted best pose with the six different DPP4 homologues studied.

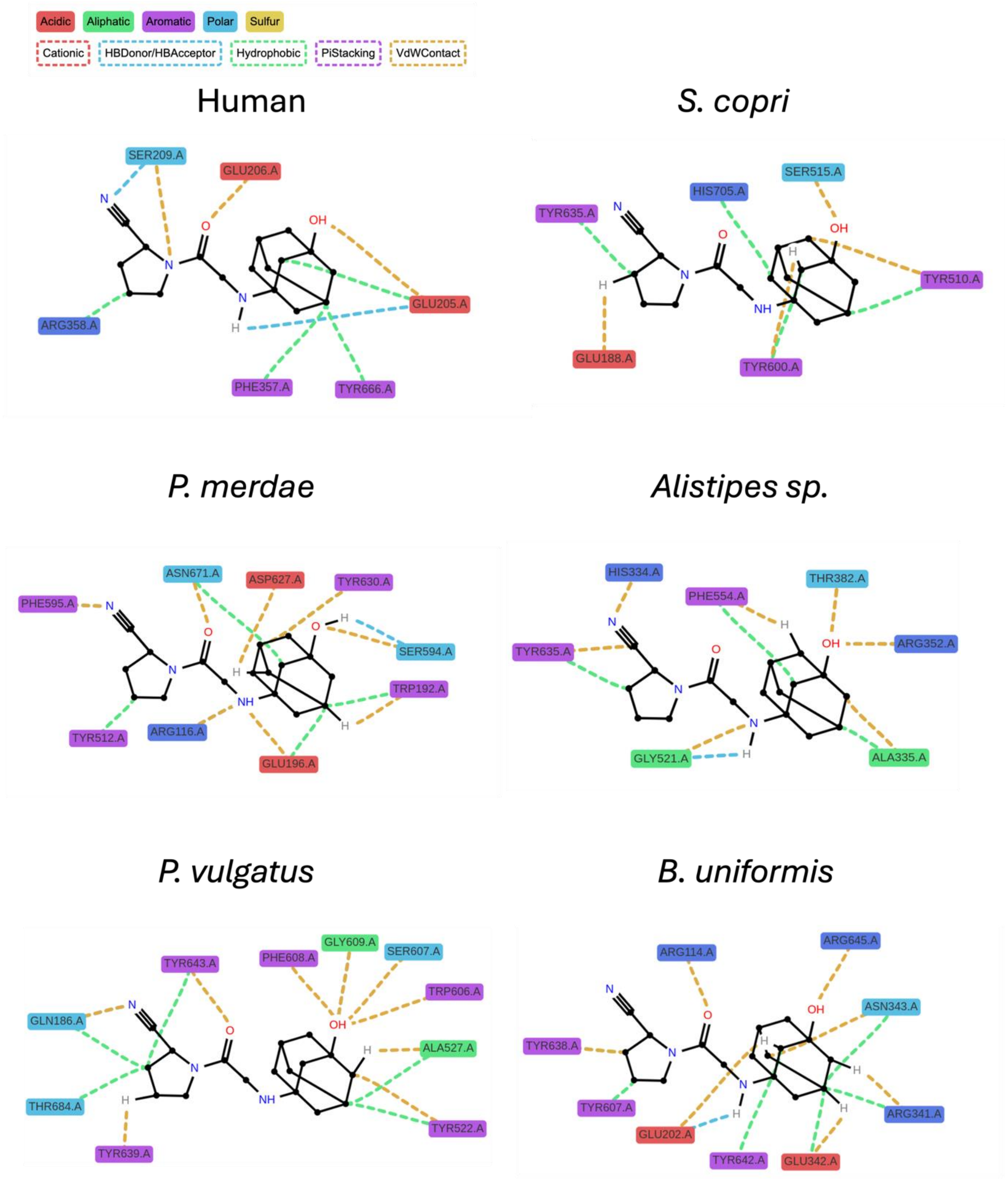

Figure S5. 2D interaction maps of vildagliptin predicted best pose with the six different DPP4 homologues studied.
